# The combined impact of mechanical factors on the wall stress of the human ascending aorta – a finite elements study

**DOI:** 10.1186/s12872-017-0733-9

**Published:** 2017-12-20

**Authors:** Tomasz Plonek, Malgorzata Zak, Karolina Burzynska, Bartosz Rylski, Anna Gozdzik, Wojciech Kustrzycki, Friedhelm Beyersdorf, Marek Jasinski, Jaroslaw Filipiak

**Affiliations:** 1 Department of Cardiac and Thoracic Surgery, Wroclaw Medical University, Borowska 213, 50-556 Wroclaw, Poland; 2 Department of Biomedical Engineering, Mechatronics and Theory of Mechanisms, Wroclaw University of Science and Technology, Wroclaw, Poland; 3 Department of Cardio-vascular Surgery, Heart Centre Freiburg University, Faculty of Medicine, University of Freiburg, Freiburg, Germany

**Keywords:** Aorta, Dissection, Aneurysm, Biomechanics, Finite elements analysis

## Abstract

**Background:**

Biomechanical factors influence stress in the aortic wall. The aim of this study was to assess how the diameter and shape of the vessel, blood pressure and longitudinal systolic aortic stretching (SAS) caused by the contraction of the myocardium influence stress in the aortic wall.

**Methods:**

Three computational models of the non-dilated aorta and aneurysms of the ascending aorta and aortic root were created. Then, finite elements analyses were carried out. The models were subjected to blood pressure (120 mmHg and 160 mmHg) and longitudinal systolic aortic stretching (0 mm, 5 mm, 10 mm and 15 mm). The influence of wall elasticity was examined too.

**Results:**

Blood pressure had a smaller impact on the stress than the SAS. An increase in blood pressure from 120 mmHg to 160 mmHg increased the peak wall stress (PWS) on average by 0.1 MPa in all models. A 5 mm SAS caused a 0.1–0.2 MPa increase in PWS in all the models. The increase in PWS caused by a 10 mm and 15 mm SAS was 0.2 MPa and 0.4 MPa in the non-dilated aorta, 0.2–0.3 MPa and 0.3–0.5 MPa in the aneurysm of the ascending aorta, and 0.1–0.2 MPa and 0.2–0.3 MPa in the aortic root aneurysm model, respectively. The loss of elasticity of the aneurysmal wall resulted in an increase of PWS by 0.1–0.2 MPa.

**Conclusions:**

Aortic geometry, wall stiffness, blood pressure and SAS have an impact on PWS. However, SAS had the biggest impact on wall stress. The results of this study may be useful in future patient-specific computational models used to assess the risk of aortic complications.

## Background

Aortic complications occur in an unpredictable way. The aortic diameter and its relation to the patient’s height and body surface area (BSA) is the main parameter taken into account when qualifying a patient for surgery. There are other factors that may increase the risk of developing acute aortic syndromes, such as genetic disorders (i.e. Marfan syndrome, Loeys-Dietz syndrome, Ehlers-Danlos syndrome), arterial hypertension, a family history of acute aortic dissection or rapid increase in the aortic diameter [9, 12].

It is believed that aortic dissection occurs when the stress in the aorta is high enough to damage the intima and allows blood flow to separate the layers of the aortic wall [20]. There are several biomechanical factors that influence the stress in the wall of the ascending aorta: the aortic wall elasticity and its tensile strength, the geometry of the vessel, the arterial blood pressure, the characteristics of blood flow and longitudinal up-and-down stretching of the aorta caused by systolic-diastolic motion of the heart [4, 10, 16, 20, 24]. However, there are no diagnostic tools that allow an objective assessment of the stress in the aortic wall and the potential risk of aortic dissection.

The aim of this study was to assess the stress distribution in the aortic root, ascending aorta and aortic arch using the finite elements method. Comparative analyses of the influence of elasticity and two dynamic factors (arterial blood pressure and longitudinal up-and-down movement of the ascending - systolic aortic stretching /SAS/) on stress in the wall of the non-dilated aorta, an aneurysm of the ascending aorta and an aneurysm of the aortic root, were also performed.

## Methods

### Model of the aorta

**Fig. 1:**
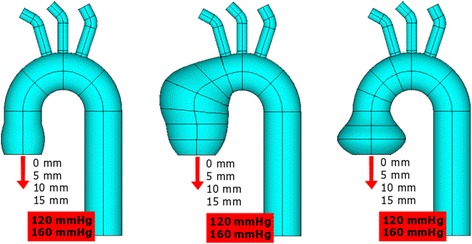
The 3D computational models of the non-dilated aorta (left), the aneurysm of the ascending aorta (middle) and the aneurysm of the aortic root (right)

Three simplified computational 3D models of a non-dilated aorta, an aneurysm of the ascending aorta and an aneurysm of the aortic root were created (Fig. 1). To make the comparison between the models more feasible, their geometries differed only in terms of the diameters of the aortic root and ascending aorta. The aorta was simulated from the level of the aortic annulus (virtual basal ring) to the descending aorta. The dimensions of the models are presented in Table 1.

**Table 1:** The parameters of the computational models

	Non-dilated aorta	Aneurysm of the ascending aorta	Aneurysm of the aortic root
Aortic root	35 mm	45 mm	55 mm
Sinotubular junction	30 mm	50 mm	40 mm
Ascending aorta	30 mm	55 mm	30 mm
Aortic arch	30 mm	30 mm	30 mm
Descending aorta	30 mm	30 mm	30 mm
Aortic arch branches	10 mm	10 mm	10 mm
Aortic wall thickness [20]	2 mm	2 mm	2 mm

### Finite elements analysis

The finite elements analyses were performed using the Ansys software (Ansys, Inc.) The 3D models of the non-dilated aorta and aortic aneurysms were divided into 244,000–293,000 3D tetrahedron SOLID187 elements with 430,000–519,000 nodes (discretization process). The elastic properties were chosen according to previously published studies [14, 16, 18]. Each aneurysm model had two versions: one, where the aortic wall elasticity was identical to the model of the non-dilated aorta (Young’s modulus: 6 MPa) and the other, where the Young’s modulus was increased up to 9 MPa. All mechanical properties of the aortic wall used in the computational models are presented in Table 2.

**Table 2:** Mechanical parameters of the aortic wall

Model	Young’s modulus	Poisson’s ratio
Non-dilated aorta	6 MPa	0,49
Aneurysm (ascending aorta and aortic root)	6 MPa, 9 MPa	0,45

The models were subjected to pressures of 120 mmHg and 160 mmHg. Moreover, a longitudinal up-and down movement of the aortic root and ascending aorta (systolic aortic stretching – SAS), caused by contraction of the heart during systole of 0 mm, 5 mm, 10 mm and 15 mm, was simulated. The distal descending aorta and distal parts of the aortic arch branches were immobilized in all directions. The distribution and maximal values of the stress (von-Mises stress) were assessed in each simulation for every model.

## Results

### Stress distribution

**Fig. 2:**
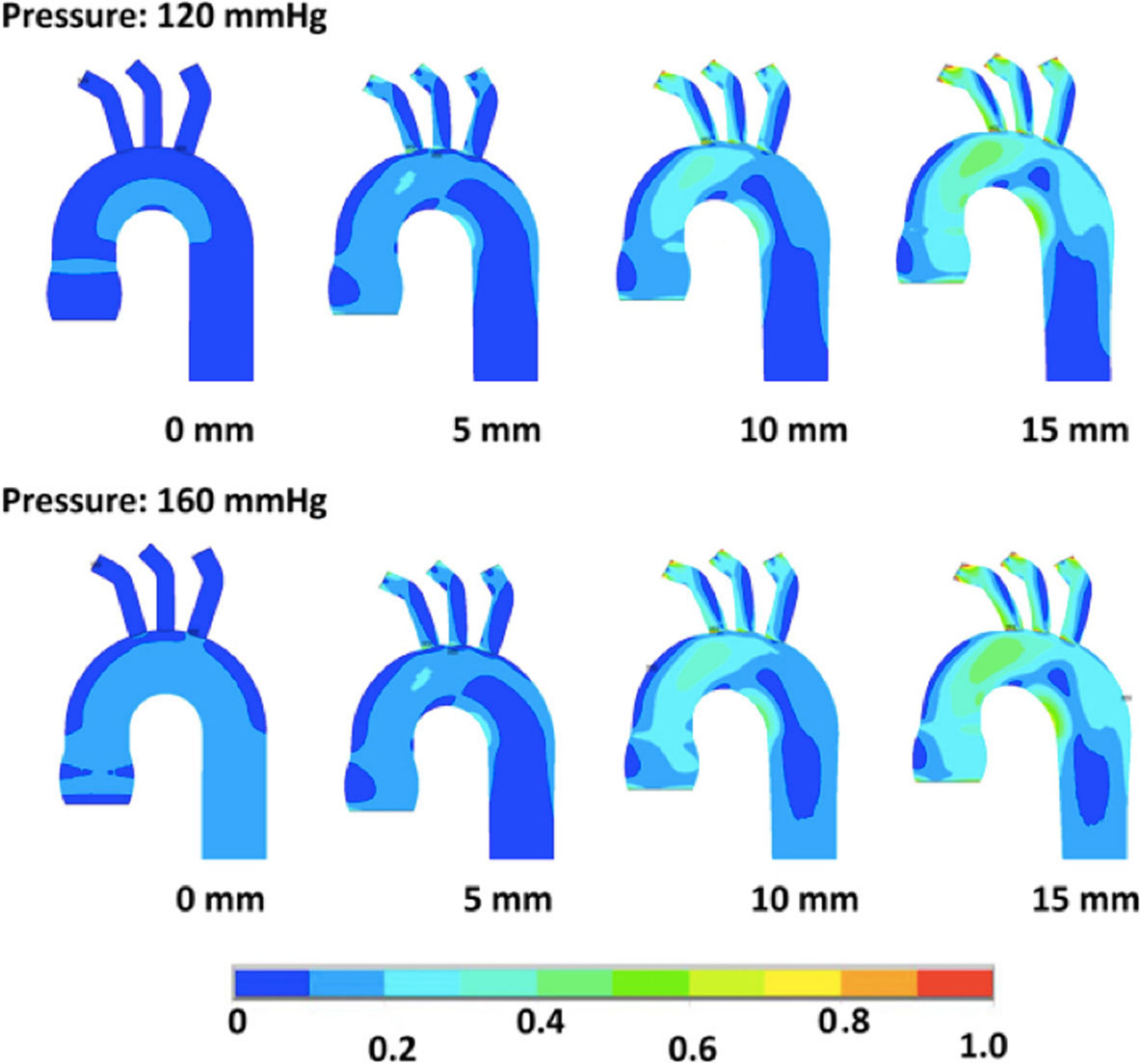
The wall stress in the model of the non-dilated aorta subjected to systolic aortic stretching of 0 mm, 5 mm, 10 mm and 15 mm. In the upper row, the models were subjected to arterial blood pressure of 120 mmHg, whereas in the lower row they were subjected to 160 mmHg. The colors represent peak wall stress in MPa

**Fig. 3:**
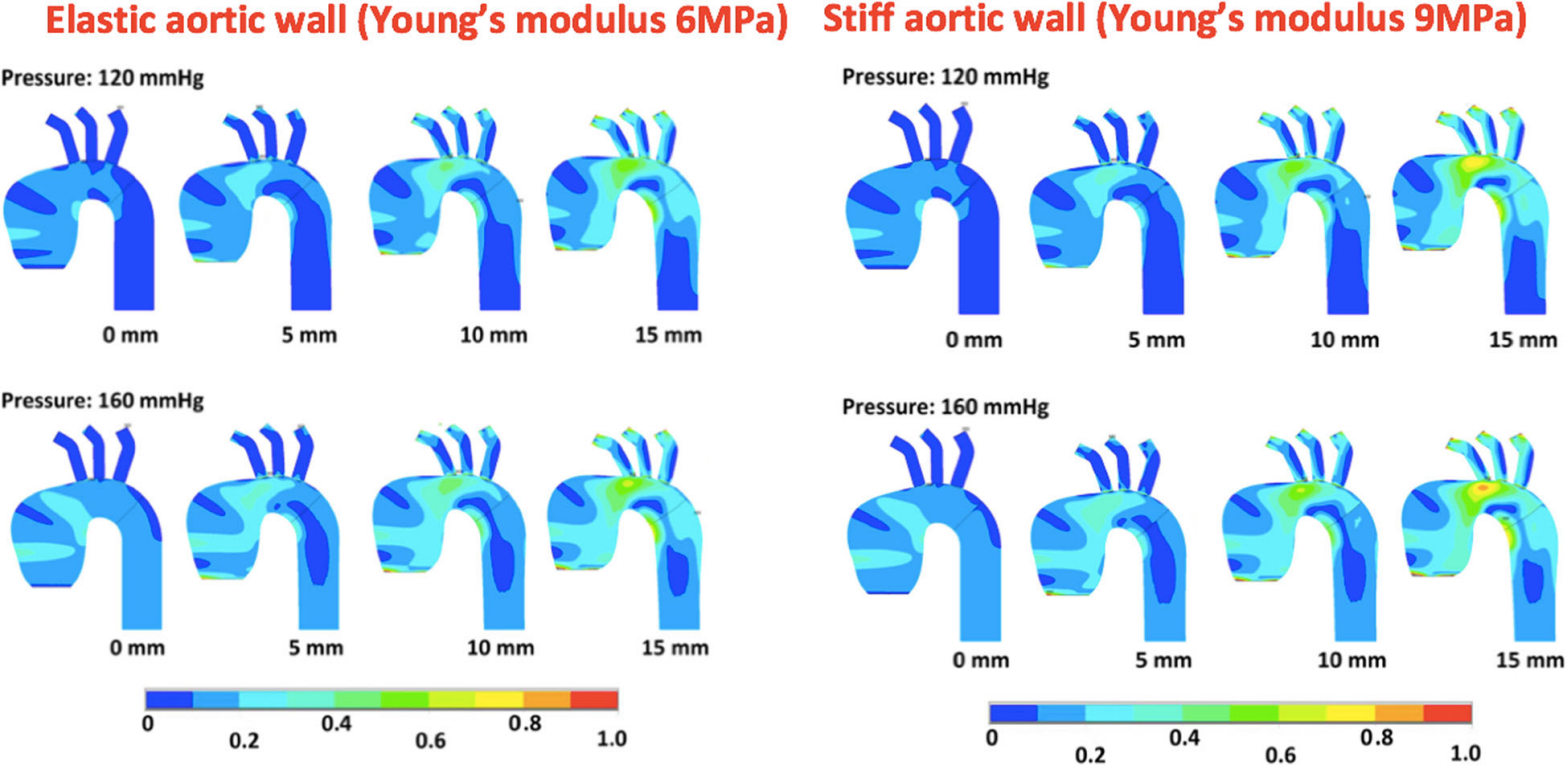
The wall stress in the model of an aneurysm of the ascending aorta subjected to systolic aortic stretching of 0 mm, 5 mm, 10 mm and 15 mm. In the upper row, the models were subjected to arterial blood pressure of 120 mmHg, whereas in the lower row they were subjected to 160 mmHg. On the left, the models have an elastic wall, whereas the models on the right have a stiff wall. The colors represent peak wall stress in MPa

**Fig. 4:**
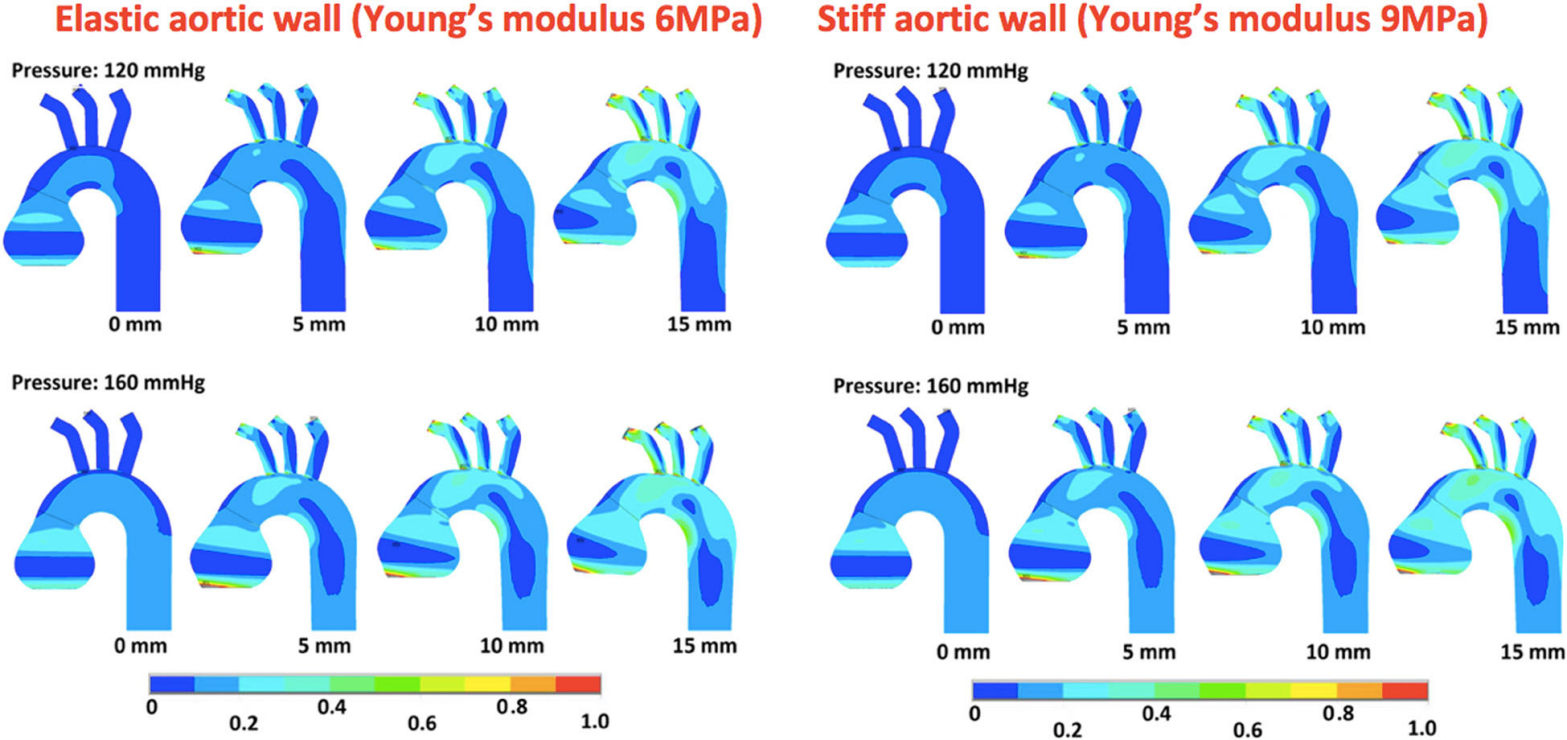
The wall stress in the model of the aortic root aneurysm subjected to systolic aortic stretching of 0 mm, 5 mm, 10 mm and 15 mm. In the upper row, the models were subjected to arterial blood pressure of 120 mmHg, whereas in the lower row they were subjected to 160 mmHg. On the left, the models have an elastic wall, whereas the models on the right have a stiff wall. The colors represent peak wall stress in MPa

The areas of the maximal stress were identified in all models. In the simulations where the systolic aortic stretching (SAS) was not applied, the maximal stress was observed in the sinotubular junction (STJ) (Figs. 2, 3 and 4). When the SAS was applied, the peak wall stress (PWS) was identified at the junction of the ascending aorta and aortic arch and in the lesser curvature between arch and descending aorta. In the ascending aorta, the stress was higher in the concavity than the convexity. The areas of the highest wall stress were not observed in the most dilated segments but at sites of abrupt change of diameter and geometry. In the aortic root model, an additional site of high stress was observed in the area of the aortic annulus. Overall, the highest PWS was observed in the model of the aneurysm of the ascending aorta with a stiff wall (Young’s modulus 9 MPa), subjected to SAS of 15 mm and blood pressure of 160 mmHg. It amounted to 0.9 MPa and was localized in the area between the ascending aorta and the aortic arch (Fig. 3).

### Arterial blood pressure

An increase in blood pressure from 120 mmHg to 160 mmHg caused an increase in the peak wall stress across the whole surface in all models. However, there was a larger increase in stress at sites of high peak stress, i.e. the sino-tubular junction or the area between the ascending aorta and the arch. The peak wall stress increased on average by 0.1 MPa regardless of the geometry, the systolic aortic stretching or the aortic wall elasticity.

### Systolic aortic stretching

Systolic aortic stretching caused a significant increase in the peak wall stress in all the models. In the non-dilated aorta, the maximal stress in the aortic wall increased with the distance to which the aorta was pulled during the systolic motion of the heart. A 5 mm increase in the SAS caused a 0.1–0.2 MPa increase in PWS in all the models. The difference in stress between models with no SAS and those with 10 mm SAS was 0.2 MPa in the non-dilated aorta, 0.2–0.3 MPa in the aneurysm of the ascending aorta, and 0.1–0.2 MPa in the aortic root aneurysm model. When the SAS was changed from 0 mm to 15 mm the PWS increased by 0.4 MPa in the non-dilated aorta, 0.3–0.5 MPa in the aneurysm of ascending aorta and 0.2–0.3 MPa in aortic root aneurysm.

### Aortic wall elasticity

The models of the aneurysms with lower aortic wall elasticity (Young’s modulus 9 MPa) had higher PWS values than those with elastic walls (Young’s modulus 6 MPa). The stress was higher on average by 0.1 MPa in models with stiffer walls. A 0.2 MPa increase in wall stress was observed in the model of the aneurysm of the ascending aorta when 15 mm systolic aortic stretching was applied.

### Geometry of the aorta

The highest wall stress was observed in the model of an aneurysm of the ascending aorta (0.9 MPa; Young’s modulus 9 MPa, SAS 15 mm, arterial blood pressure of 160 mmHg). The maximal wall stress in the models of non-dilated aorta and aortic root aneurysm were 0.5 MPa (Young’s modulus 6 MPa, SAS 15 mm, arterial blood pressure of 160 mmHg) and 0.5 MPa (Young’s modulus 9 MPa, SAS 15 mm, arterial blood pressure of 160 mmHg). The stress distribution differed between models, although some areas of PWS were similar, i.e. the sino-tubular junction and the area between the aortic arch and the ascending aorta. Moreover, the aortic root model had higher stress in the area of the ventriculo-aortic junction (0.5 MPa) compared to the non-dilated aorta (0.3 MPa) and the aneurysm of the ascending aorta (0.3 MPa).

## Discussion

There is no objective method to assess the risk of development of an aortic dissection. According to a recent study, type A aortic dissection usually occurs when the diameter of the vessel is approximately 40 mm [26]. This is much lower than the threshold for qualifying the patient for surgery [9, 12]. Therefore, a new method to estimate the risk of dissection, which takes into account all currently known risk factors, is necessary. We believe that a finite elements analysis could be used to define this risk for an individual patient [16, 20].

Biomechanical factors play a crucial role in the process of aortic dissection [21]. It is believed that blood pressure, the diameter and elasticity of the aorta have the most significant impact on stress in the aortic wall [4, 6, 7, 9, 21, 27]. However, it is still unknown which factor has the biggest influence on mechanical stress. The geometry of the proximal ascending aorta varies significantly between individuals [8] and therefore a multifactorial assessment is necessary to estimate the stress in the aortic wall. Several studies assessed the influence of biomechanical parameters on the stress of the wall of the ascending aorta, including wall stiffness [4, 16, 20], aortic root motion [4], blood pressure [4, 10, 20, 24], flow shear stress [24], the geometry of the aorta and aortic valve type [1, 5, 11, 17, 22, 23, 25]. However, there are no studies assessing the wall stress in an aneurysm model subjected to aortic root motion. Moreover, no comparative analysis of stress distribution between a non-dilated aorta, aortic root aneurysm and an aneurysm of the ascending aorta has been performed. To date, this is the first study to perform complex biomechanical analyses assessing the impact of all currently known biomechanical factors responsible for aortic stress and comparing various types of aortic geometries.

The factor that significantly influences stress in the ascending aorta is longitudinal stretching of the aorta caused by systolic movement of the heart (systolic aortic stretching, SAS) [4, 25]. Based on a study that assessed magnetic resonance images (MRI) of 11 healthy patients, the SAS is on average 8.9 mm [15]. In the Reykyavik study, aortic annulus motion assessed by MRI in 347 patients over 70 years of age was 6.8 mm in men and 7.8 mm in women [3]. According to Beller and colleagues, an 8.9 mm displacement of the aortic annulus resulted in a stress increase comparable to the rise in blood pressure by 60 mmHg (from 120 mmHg to 180 mmHg). One of the most common sites for the entry of type A aortic dissection is the transverse tear near the sinotubular junction [13]. A transverse tear can occur due to the longitudinal stretch of the aortic wall. Such a longitudinal stretch is caused by SAS [4, 25]. Based on our results, SAS has a bigger impact on aortic wall stress than blood pressure. A 10 mm SAS caused a rise in wall stress two times higher than a 40 mmHg increase in blood pressure. This finding supports the thesis that a positive inotropic effect of some drugs may increase the risk of dissection [2] and certain negative inotropic drugs, i.e. beta blockers can reduce this risk [28].

The MRI studies in healthy subjects revealed a slight axial twist of 6–14 degrees of the ascending aorta during systole [29]. However, this did not impact the wall stress [4] and thus, was not simulated in our model. Blood flow shear stress in the ascending aorta was estimated at the level of around 0.0023 kPa. This is several orders of magnitude lower than the stress caused by blood pressure and may influence the growth of the vessel over years, but is not likely to cause an intimal tear and be a direct cause of a dissection. Therefore, flow shear stress was not assessed in our analyses [24].

The maximal wall stress was observed in the sinotubular junction (STJ) area, at the junction of the ascending aorta and aortic arch and in the lesser curvature between the arch and the descending aorta. These areas correlate with the most common sites of entry in type A aortic dissection [13]. In the aortic root model, an additional zone of high stress was observed in the aortic annulus. This finding may explain why aortic root aneurysms are often accompanied by annular dilatation.

The PWS was not observed in the areas where the diameter was large, but at sites of an abrupt change in the vessel geometry, i.e. the sinotubular junction. This finding is consistent with other studies, which report PWS to be located in areas of sudden morphological change, i.e. aneurysm necks [19, 22, 27].

The aortic wall stiffness had a significant influence on the wall stress. The aneurysm models with stiffer walls had higher peak stress by 0.1–0.2 MPa compared to those of normal elasticity. This was comparable to the stress caused by a 40 mmHg increase in systolic blood pressure.

**Fig. 5:**
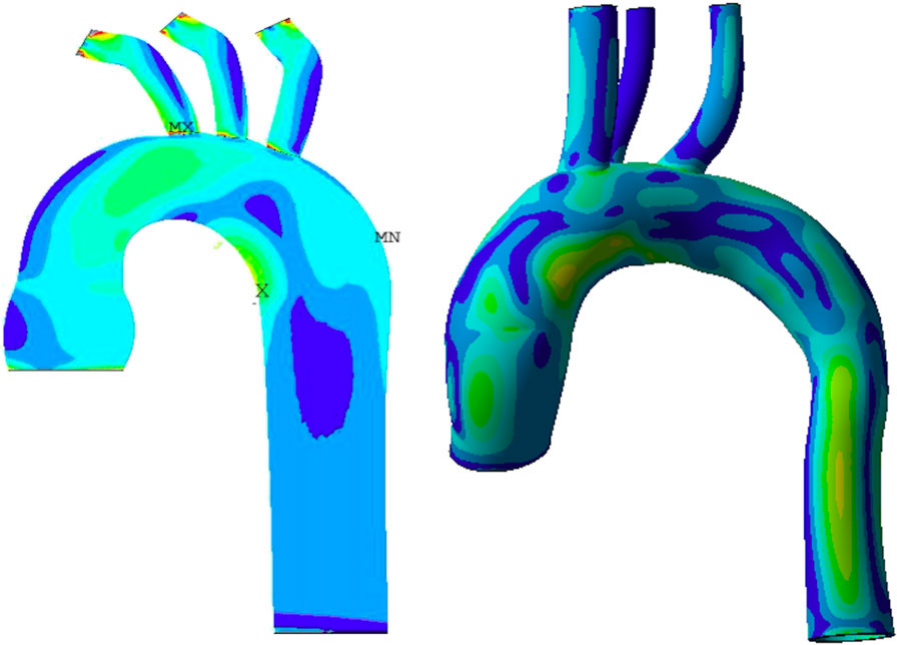
A comparison between the patient-specific and the idealized model of the thoracic aorta

The geometry of the aortic model had a significant impact on the peak wall stress and stress distribution. The highest PWS was observed in the model of an aneurysm of the ascending aorta. However, to estimate wall stress correctly, a patient-specific model is necessary. Such simulations on patient-specific models with the aortic wall composed of three layers have already been performed by our group. The stress values and stress distribution was similar in both - patient-specific and the idealized models (Fig. 5). Nevertheless, some differences between the models were observed. Therefore, further studies are necessary to estimate what an acceptable simplification of the aortic model is. Current diagnostic tools are not able to define local aortic wall mechanical properties. In the near future, high resolution MRI scanners may be able to assess the local thickness and elasticity of the aorta with a resolution which will allow for reliable computational numerical reconstructions.

### Study limitations

The aortic wall is an inhomogeneous layered structure with nonlinear anisotropic mechanical properties [10, 30]. To date, there are no examinations allowing for an exact in vivo assessment of the biomechanical properties of the wall of the ascending aorta and aortic aneurysm. Therefore, a simplified model with linear isotropic properties of the aortic wall was used. Nevertheless, in future studies, the patient specific material properties and aortic geometry must be taken into account to evaluate stress correctly [16, 20]. The fluid-structure interaction was not used because this type of simulation made it impossible to implement the movement of the aortic annulus to mimic the stretching of the aorta. This study is a theoretical view, which is more an indication that longitudinal stretching of the aorta should be implemented in the simulations of the thoracic aorta than a reliable estimation of the real values of stress in the thoracic aorta.

## Conclusions

The results of this study may be useful in future patient-specific computational models used to assess the risk of aortic complications. Our results help to differentiate how individual factors influence wall stress. Systolic aortic stretching in the ascending aorta is a phenomenon that cannot be omitted when performing stress analyses. SAS had a larger impact on the wall stress than blood pressure, wall stiffness and the geometry of the vessel. Moreover, the sites of peak wall stress correlate with typical areas of dissection entry.

## Abbreviations

3D: Three-dimensional
BSA: Body surface area
MRI: Magnetic resonance imaging
PWS: Peak wall stress
SAS: Systolic aortic stretching
STJ: Sino-tubular junction
